# Active children are less adipose and insulin resistant in early adolescence; evidence from the Mysore Parthenon Cohort

**DOI:** 10.1186/s12887-019-1855-2

**Published:** 2019-12-18

**Authors:** Sarah H. Kehoe, Ghattu V. Krishnaveni, Sargoor Veena, Krishnarajasagara N. Kiran, Samuel C. Karat, Asha Dhubey, Patsy Coakley, Caroline H. D. Fall

**Affiliations:** 1 Medical Research Council Lifecourse Epidemiology Unit, Southampton General Hospital, Tremona Road, Southampton, SO16 6YD UK; 2 Epidemiology Research Unit, Holdsworth Memorial Hospital, Mandi Mohalla, Mysuru, Karnataka 570021 India

**Keywords:** Adolescent, Cardiometabolic risk, Children, India, Insulin resistance, Physical activity

## Abstract

**Background:**

The aim of this study was to determine whether physical activity volume and intensity in mid-childhood and early adolescence were associated with cardiometabolic risk factors at 13.5 years.

**Methods:**

Participants were recruited from the Mysore Parthenon observational birth cohort. At ages 6–10 and 11–13 years, volume and intensity of physical activity were assessed using AM7164 or GT1M actigraph accelerometers worn for ≥4 days, and expressed as mean counts per day and percentage time spent in light, moderate and vigorous physical activity according to criteria defined by Evenson et al. At 13.5 years, fasting blood samples were collected; lipids, glucose and insulin concentrations were measured and insulin resistance (HOMA) was calculated. Systolic and diastolic blood pressure were measured at the left arm using a Dinamap (Criticon). Anthropometry and bio-impedance analysis were used to assess body size and composition. Metabolic and anthropometric measures were combined to produce a metabolic syndrome risk score.

**Results:**

At 6–10 years, boys and girls respectively spent a median (IQR) of 1.1 (0.5, 2.0) % and 0.8 (0.4, 1.3) % of recorded time vigorously active. At 11–13 years, boys and girls respectively spent a median (IQR) of 0.8 (0.4, 1.7) % and 0.3 (0.1, 0.6) % of time vigorously active. All of the physical activity parameters were positively correlated between the 6–10 year and the 11–13 year measurements indicating that physical activity tracked from childhood to early adolescence. There were no associations between physical activity at 6–10 years and individual 13.5 year risk factors but % time vigorously active was inversely associated with metabolic syndrome score (B = −0.40, 95% CI −0.75, 0.05). Volume of physical activity at 11–13 years was inversely associated with 13.5 year HOMA and fat percentage and vigorous physical activity was associated with HOMA, fat percentage, sum of skinfolds, waist circumference and total: HDL cholesterol ratio. Vigorous physical activity was inversely associated with metabolic syndrome score (B = −0.51, 95% CI −0.94, −0.08).

**Conclusions:**

Volume and intensity of physical activity in early adolescence were negatively associated with metabolic and anthropometric risk factors. Interventions that aim to increase adolescent physical activity, especially vigorous, may prevent cardiometabolic disease in later life.

## Background

Cardiovascular disease (CVD) was the leading cause of death and disability globally in 2017 [1]. It has been estimated that in India deaths attributable to cardiovascular causes will increase from 2.7 million in 2004 to 4 million in 2030 [2]. Diabetes is a risk factor for CVD and in 2015 worldwide diabetes prevalence was estimated at 415 million. It is predicted to increase to 642 million by 2040 [3].

There is heterogeneity between middle income countries in terms of the burden of cardiometabolic disease. However, three quarters of people with diabetes live in low- and middle-income countries and there is a particularly high burden in South Asia. For example, 9% of the Indian population aged 20–79 have type 2 diabetes and there is evidence that there is a further proportion who are undiagnosed or have impaired glucose tolerance [3]. The increase in childhood overweight and obesity [4–6] is thought to contribute to the increase in cardiometabolic disease prevalence in India.

There is evidence from studies in South Asia and among children of South Asian origin living in the UK that excess body fat and abdominal adiposity are risk factors for insulin resistance and type 2 diabetes [7]. In addition, insulin metabolism among children of South Asian origin living in the UK appears to be more sensitive to adiposity than among their white Caucasian counterparts [8]. The effect of adiposity on insulin metabolism appears to occur earlier in childhood among those of South Asian origin [9]. These findings indicate that South Asian children and adolescents may be at particularly high risk of becoming insulin resistant and developing type 2 diabetes in later life.

Insufficient physical activity has been suggested as one of the causes of the increase in cardiometabolic disease in India [2]. There is some evidence from European and North American studies that increased physical activity is associated with reduced risk factors for cardiometabolic disease including insulin resistance [10–12] and that sedentary time is positively associated with cardiometabolic risk [13]. There is inconsistent evidence on the association between physical activity and blood pressure and blood lipids in childhood [14]. Research in a European cohort found an inverse association between vigorous physical activity in childhood and a composite score of cardiometabolic disease risk in adolescence [15].

Physical activity, as a behaviour has been found to track from childhood into adulthood with adolescence being a critical time for maintenance of physical activity particularly for girls [16, 17]. It is thought that girls may become less active in adolescence due to onset of menarche and cultural factors that make physical activity less desirable. Interventions that can address these factors and encourage adolescents to maintain physical activity may be helpful in preventing cardiometabolic disease. A recent study investigated several components of physical activity behaviour and infrastructure among 49 countries and found that there was variability in physical activity levels among children and adolescents. In general, middle income countries scored lower than high income countries with India being ranked in the bottom 10 out of 49 [18].

To our knowledge no studies have investigated longitudinal associations between objectively measured physical activity and risk factors for later cardiometabolic disease among children or adolescents in India. Our objectives were to study (1) trends in physical activity with age and (2) longitudinal associations between physical activity in childhood and early adolescence and risk factors including insulin resistance, body fat percentage, skinfold thicknesses, waist circumference, waist hip ratio, blood lipids and blood pressure at 13.5 years in a South Indian birth cohort. Our hypothesis was that participants who were more physically active in childhood and early adolescence would be at lower risk of cardiometabolic disease at 13.5 years.

## Methods

### Study design and setting

We used longitudinal data from an observational birth cohort study named the Mysore Parthenon Birth Cohort [19]. The study was conducted in and around the city of Mysore in the South Indian state of Karnataka. The current study was a secondary analysis of data collected as part of the birth cohort study.

### Participants

The participants were born to women recruited into the Mysore Parthenon study. In 1997–1998 pregnant women living in the city of Mysore and surrounding rural areas were recruited to the study if they fulfilled the following criteria: non-diabetic prior to pregnancy; <32 weeks gestation at time of recruitment; planning to deliver at Holdsworth Memorial Hospital (HMH) and had a singleton pregnancy. Babies were included in the study if they had no major congenital anomalies. Full details of the cohort have been published previously [20]. In brief, 663 women receiving care at the ante-natal clinic of HMH, Mysore, South India gave birth to live singleton babies. Detailed anthropometry of the offspring was obtained within 72 h of birth and they were followed up every 6 months thereafter.

**Fig. 1:**
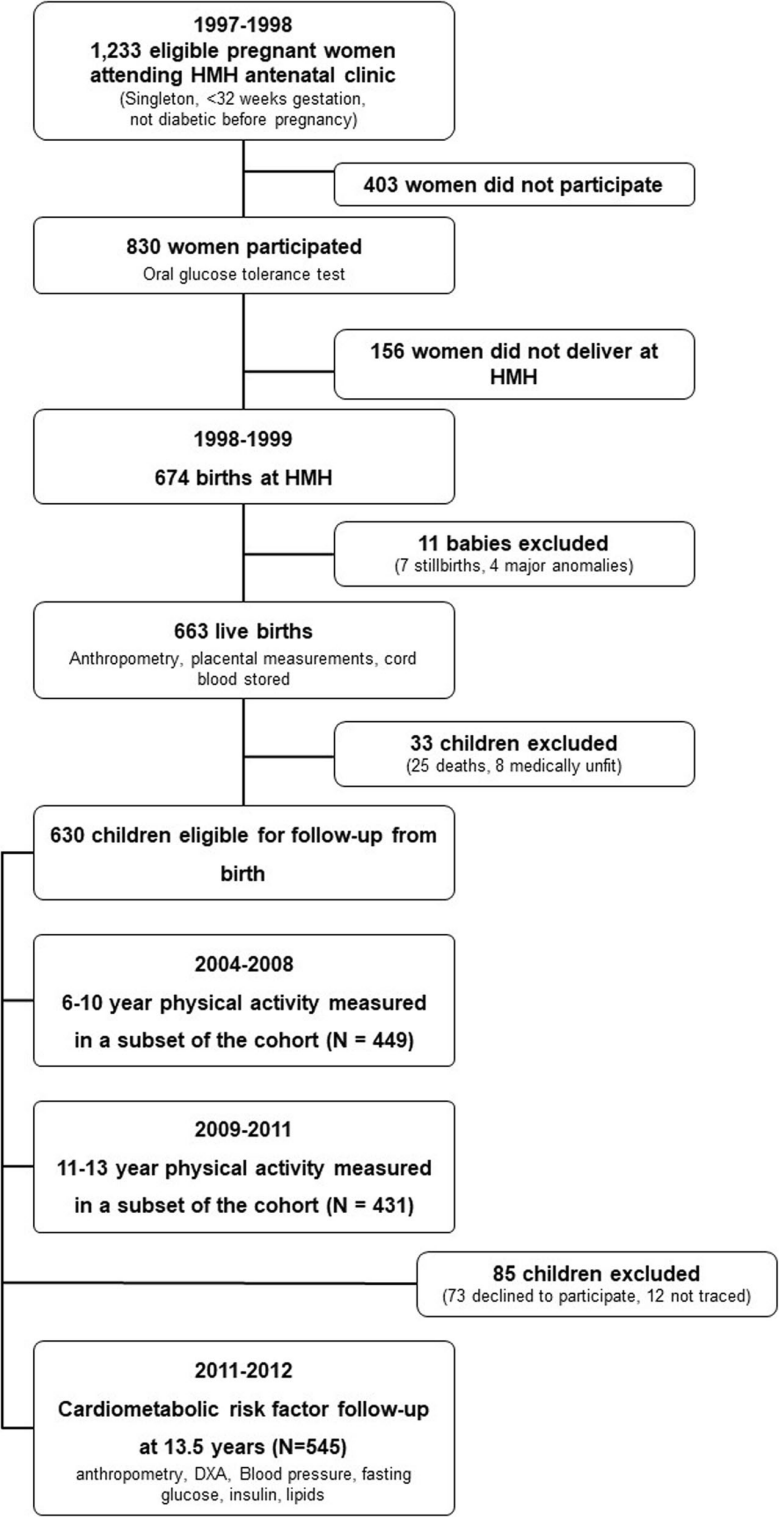
Participant flow chart

We asked a subset of the participants to wear accelerometers when they were aged between 6 and 10 years and again at age 11–13 years. When the participants were aged exactly 13.5 years we collected data on risk factors for cardiometabolic disease. Figure 1 shows the flow of participants through the study.

The study was conducted according to the standards of the Declaration of Helsinki. Ethical permission for the study was obtained from the Holdsworth Memorial Hospital Ethics Committee, Mysore. Parents gave informed consent and participants gave assent to take part in the study.

### Physical activity

Accelerometers measure accelerations in a vertical plane and yield data as counts. Counts are used as a proxy for the volume of physical activity the wearer engages in. The volume of physical activity is the product of intensity and duration of physical activity.

A convenience sample of children in the cohort were asked to wear the AM7164 or GT1M actigraph accelerometer (MTI Health Services, Florida, USA) for 7 days at two timepoints, once between the ages of 6–10 years and once between the ages of 11–13 years. The accelerometers weighed approximately 38 g and were worn at the right hip attached to an elastic belt, they were not waterproof so had to be removed during bathing and water-based activities. The child put the accelerometer on after getting up in the morning and removed it at night before going to bed. Following the measurement period, the accelerometers were returned and the data downloaded and processed using Mahuffe software (MRC Epidemiology Unit, Cambridge, UK). A correction factor was used to ensure that data from the two types of accelerometer was comparable [21] in terms of total counts and counts per minute (cpm).

### Cardiometabolic risk factors

All risk factor measurements were made when the participants were exactly 13.5 years old.

#### Size and body composition

The following measurements were made by trained measurers: weight to the nearest 0.1 kg (Salter digital scales, UK); height to the nearest 0.1 cm (Microtoise, CMS Instruments, UK); waist circumference to the nearest 0.1 cm; triceps and subscapular skinfolds to the nearest 0.1 mm (Harpenden callipers, CMS Instruments). The inter-observer coefficient of variation (CV) was <1% for height and weight, 1.3% for triceps and 4.5% for subscapular skinfold thickness.

Bio-impedance analysis was carried out using Bodystat Quadscan 4000 and 1500MDD machines (Bodystat, UK) in order to estimate percentage body fat. Any metal jewellery was removed and participants were asked to lie supine for 5 min before the measurements. After cleaning the skin with surgical spirit, one electrode was attached at the level of the ulnar head at the wrist and the other just behind the knuckles. On the foot, the two electrodes were attached at the level of the medial and lateral malleoli and behind the toes, respectively. This method has been shown to give reliable estimates of fat and fat-free mass [22, 23]. We have observed in a subgroup of the cohort that bio-impedance was useful for measuring group-level changes in adiposity [24].

#### Insulin resistance and lipid profile

Glucose, insulin and lipid concentrations (HDL cholesterol, LDL cholesterol and triglycerides) were measured using fasting blood samples. The laboratory assays were carried out at the Diabetes Unit, KEM Hospital, Pune, India, a member of the UK National External Quality Assessment Service (NEQAS) quality control programme for insulin assays. Laboratory staff were blind to the identity of the samples. Plasma glucose and lipid concentrations were measured by standard enzymatic methods (Hitachi-902; Roche Diagnostics, Mannheim, Germany). Insulin was measured by ELISA (Mercodia Ultrasensitive; Mercodia, Uppsala, Sweden). Inter- and intra-assay CV was <7.0%. Insulin resistance was estimated using Homeostasis Model Assessment for insulin resistance (HOMA-IR) [25].

#### Blood pressure

Systolic and diastolic blood pressure (BP) were measured using an automated BP monitor (Dinamap 8100, Criticon, FL, USA).

#### Metabolic syndrome score

We used the published method of Eisenmann [26] to calculate a metabolic syndrome score in order to obtain a composite score of cardiometabolic risk. The score was the sum of the following variables (all Z scores with a mean of 0 and a standard deviation of 1): waist circumference, mean arterial pressure,^1^ HOMA-IR, triglycerides, ratio of total cholesterol to HDL cholesterol. A higher score indicated greater cardiometabolic risk.

### Covariates

#### Gestational diabetes

The mothers of the participants underwent a 100 g, 3 h oral glucose tolerance test at 30 ± 2 weeks gestation. Gestational diabetes was defined using Carpenter and Coustan criteria [27].

#### Demographic data

An interviewer-administered questionnaire was used to collect information about socio-economic status using the NFHS-2 Standard of Living Index [28]. The respondent was a parent or close relative.

#### Pubertal staging

Pubertal status was assessed at 13.5 years by a trained member of the research team using Tanner’s method [29], and was classified as the stage of breast development (girls) or genital development (boys).

### Data analysis

Variables that did not approximate a normal distribution were log transformed for analysis. We used independent t tests to study the differences between males and females in terms of physical activity and risk factors for cardiometabolic disease.

#### Physical activity

The accelerometers were programmed to collect data in 1 min epochs. It was assumed that the monitors had been taken off if zero counts were recorded for a period of more than 20 min. Therefore registered time was defined as all time during which counts were recorded with gaps of no more than 20 min of consecutive zero cpm. The first and final days of actigraph wearing were not included in the data analysis. Days with less than 500 min of registered time were considered to be unrepresentative of the participant’s physical activity pattern and therefore excluded from further analysis. Allowing for this at least 4 days of data were available for all of the participants. The physical activity variables were in the form of total counts/day and proportion of time spent at different levels of intensity (sedentary, light, moderate and vigorous).

We multiplied counts per day derived from the AM7164 device by 0.91 to allow these variables to be comparable with data from the GT1M device [21]. We present results based on cut offs developed by Evenson et al. [30] <100 cpm for ‘sedentary behaviour’, 100–2291 cpm for ‘light physical activity’; 2292–4008 cpm for ‘moderate physical activity’; >4008 cpm for ‘vigorous physical activity’ (V). These categories have been shown to classify physical activity more precisely in children than other published cut offs [31].

#### Bio-impedance

Fat percentage values from impedance measurements at 50 kHz were generated using the manufacturer’s equation which included terms for sex, age, height and weight.

#### Physical activity tracking

We calculated partial Pearson correlation coefficients adjusted for sex and age at first physical activity monitoring to study tracking at the individual level between the two time points at which physical activity was measured.

#### Statistical procedures

We used univariate and multivariate linear regression models to study the association between physical activity at both time points and cardiometabolic risk factors at 13.5 years. In the multivariate model we included the following covariates: age at physical activity assessment, sex, pubertal stage at 13.5 years, standard of living, maternal gestational diabetes status.

The data were analysed using SPSS software package version 21 (SPSS Inc., Chicago, IL).

## Results

Between the ages of 6 and 10 years, 449 participants agreed to physical activity monitoring with an accelerometer. Between the ages of 11 and 13 years, 431 agreed to monitoring. At 13.5 years, data were collected from 545 participants on the risk factors for cardiometabolic disease. Of these 545, physical activity data at both time points were available for 290 (Fig. 1).

Table 1 shows the physical activity data collected at both time points by gender. Males were more physically active than females in terms of total volume of physical activity and moderate and vigorous physical activity at both time points. Sedentary time and light physical activity did not differ between sexes at 6–10 years.

**Table 1:** Physical activity measures at 6–10 years and 11–13 years*

	6–10 years			11–13 years		
Physical Activity measures*	Males	Females		Males	Females	
	*n* = 140	*n* = 150	*p*^**§**^	*n* = 175	*n* = 194	*p*^**§**^
Counts/ day (× 10^3^)	448 ± 128	381 ± 110	< 0.01	418 ± 144	296 ± 101	< 0.01
Registered Time (min/d)	798 ± 81	783 ± 77	0.11	778 ± 86	760 ± 74	0.03
Sedentary (% of RT) ^†^	40.7 ± 9.6	42.6 ± 9.5	0.51	47.9 ± 11.1	53.6 ± 10.0	< 0.01
Light physical activity (% of RT)^†^	53.0 ± 8.3	52.8 ± 8.7	0.38	46.2 ± 9.6	43.2 ± 9.2	< 0.01
Moderate physical activity (% of RT)^†^	5.0 ± 2.2	3.6 ± 1.7	< 0.01	4.7 ± 2.5	2.8 ± 1.6	< 0.01
Vigorous physical activity (% RT)^†^	1.1 (0.5, 2.0)	0.8 (0.4,1.3)	< 0.01	0.8 (0.4,1.7)	0.3 (0.1,0.6)	< 0.01

*Data are expressed as mean ± SD for normally distributed variables and as median (IQR) for non-normally distributed variables. ^†^Intensity cut offs are those published by Evenson [30]; *CPM* Counts per minute, *RT* registered time ^**§**^ *p* value refers to t test of difference between males and females

Table 2 shows that there was a correlation between physical activity volume and intensity at both time points indicating that physical activity tracks with age in this population. The correlation was stronger for total volume of physical activity, sedentary time and light physical activity than for moderate and vigorous physical activity indicating that the latter two may not track as closely. This could be due to the relatively low participation in moderate and vigorous activity in this population.

**Table 2:** Tracking of physical activity between childhood and early adolescence; partial Pearson’s correlation adjusted for sex and age at 6–10 year measurement (*n* = 290)

	11–13 year Physical activity measures					
		Counts/ day	% time Sedentary	% time Light	% time Moderate	% time Vigorous
6–10 year Physical activity measures	Counts/day	0.32**	−0.24**	0.20*	0.26**	0.18*
	% time Sedentary	−0.30**	0.35**	−0.33**	−0.23**	− 0.06
	% time Light	0.26**	−0.34**	0.34**	0.18*	0.01
	% time Moderate	0.24**	−0.19*	0.14*	0.26**	0.14*
	% time Vigorous	0.13*	−0.07	0.03	0.14*	0.20*

*p for correlation <0.05, **p for correlation <0.001

Anthropometric and cardiometabolic disease risk factor data at 13.5 years are presented in Table 3. There were no sex differences in terms of height but females were heavier and had higher body fat percentage and skinfold thickness measurements than males. Females also had a larger waist circumference but smaller waist to hip ratio. In terms of risk factors, females had lower blood pressure, higher insulin and HOMA, and higher total and LDL cholesterol and triglycerides.

**Table 3:** Body composition and chronic disease risk factor measurements at 13.5 years*

	Males (*n* = 175)	Females (*n* = 194)	*p*^§^
Measures at 13.5y			
Weight (kg)	40.8 ± 8.3	43.5 ± 8.4	< 0.01
Height (cm)	154.4 ± 8.2	153.5 ± 5.9	0.22
BMI (kg/m^2^)	16.4 (15.4,18.0)	17.9 (16.2, 20.3)	< 0.01
Waist circumference (cm)	65.5 ± 7.7	67.7 ± 7.9	< 0.01
Waist Hip Ratio	0.89 ± 0.05	0.87 ± 0.05	< 0.01
MUAC (cm)	21.9 ± 2.7	22.3 ± 2.7	0.07
Triceps skin-fold (mm)	9.6 (7.7, 14.1)	14.5 (11.2, 18.4)	< 0.01
Subscapular skin-fold (mm)	9.2 (7.2, 13.6)	15.3 (11.3, 20.3)	< 0.01
Sum of skinfolds (mm)	18.3 (15.0, 28.6)	29.4 (22.3, 39.5)	< 0.01
Fat % from bio-impedance	17.6 ± 6.9	26.1 ± 5.8	< 0.01
Systolic blood pressure	110.5 ± 8.9	107.9 ± 7.9	< 0.01
Diastolic blood pressure	62.3 ± 7.9	58.8 ± 6.8	< 0.01
Fasting glucose (mmol/L)	5.07 ± 0.53	5.02 ± 0.48	0.39
Fasting insulin (pmol/L)	33.7 (22.4, 48.8)	46.1 (36.5, 64.4)	< 0.01
HOMA-IR	1.27 (0.82, 1.80)	1.76 (1.35, 2.38)	< 0.01
Total cholesterol (mmol/L)	3.45 ± 0.69	3.59 ± 0.62	0.04
HDL cholesterol (mmol/L)	1.07 ± 0.25	1.06 ± 0.23	0.52
LDL cholesterol (mmol/L)	2.01 ± 0.55	2.13 ± 0.48	0.04
Triglycerides (mmol/L)	0.69 (0.49, 0.98)	0.79 (0.60, 1.07)	0.02

*Values are mean ± SD for normally distributed variables and median (inter-quartile range) for non-normally distributed variables. ^**§**^ *p* value refers to t test of difference between males and females. *MUAC* Mid-upper arm circumference, *HOMA* Homeostasis Model Assessment for insulin resistance

Univariate regression models indicated that all measures of physical activity at 6–10 years were negatively associated with body fat percentage (Table 4). These associations were all attenuated in the adjusted models (Table 5). Total counts per day were negatively associated with insulin resistance, body fat percentage, sum of skinfolds and waist circumference in univariate models but only the associations with insulin resistance and body fat percentage remained after adjustment. Moderate physical activity was negatively associated with fat % and waist circumference in both the unadjusted and adjusted models. Vigorous physical activity was negatively associated with insulin resistance, fat percentage, sum of skinfolds, waist circumference and total: HDL cholesterol ratio in both univariate and multivariate models. In the multivariate models, there was a negative association between vigorous physical activity at 6–10 years and the composite metabolic syndrome score, likewise moderate and vigorous physical activity at 11–13 years were inversely associated with the metabolic syndrome score.

**Table 4:** Univariate linear regression models with physical activity measures as independent variables and 13.5 year cardiometabolic risk factors as the dependent variables (*n* = 290)

	13.5y Risk Factors							
	HOMA-IR	Fat %	Sum of Skinfolds (mm)	Waist Circumference (cm)	Total:HDL Cholesterol	Triglycerides	SBP (mmHg)	Metabolic Syndrome Score^§^
	B (95% CI)							
6–10 year Physical Activity								
Counts/day × 10^3^	0.00	−0.01*	0.00	0.00	0.00	0.00	0.00	0.01
	(−0.01, 0.01)	(−0.02,−0.01)	(−0.01,0.01)	(−0.01,0.01)	(−0.01,0.01)	(−0.01,0.01)	(−0.01,0.01)	(−0.02,0.03)
% time Moderate	−0.04	−0.57*	0.03	0.02	0.02	0.03	−0.01	0.08
	(−0.10, 0.03)	(−1.00,−0.15)	(−0.03,0.09)	(−0.03,0.08)	(−0.04,0.07)	(−0.03,0.08)	(−0.06,0.05)	(−0.10,0.25)
% time Vigorous	−0.07	−0.97*	−0.02	−0.01	−0.10	−0.08	−0.04)	−0.20
	(−0.20, 0.06)	(−1.87,−0.08)	(−0.14,0.10)	(−0.12,0.11)	(−0.22,0.02)	(−0.20,0.04)	(−0.16,0.08)	(−0.55,0.15)
11–13 year Physical Activity								
Counts/day × 10^3^	−0.01*	−0.02**	−0.01*	−0.01*	0.00	0.00	0.00	−0.01
	(−0.02,0.00)	(−0.03,−0.01)	(−0.02,0.00)	(−0.02,0.00)	(−0.01,0.01)	(−0.01,0.01)	(−0.01,0.00)	(−0.03,0.02)
% time Moderate	−0.04	−1.17**	−0.04	−0.05*	−0.01	−0.01	−0.01	−0.03
	(−0.08,0.10)	(−1.49,−0.85)	(−0.09,0.00)	(−0.09,0.00)	(−0.06,0.03)	(−0.05,0.04)	(−0.05,0.04)	(−0.17,0.10)
% time Vigorous	−0.14*	−2.74**	−0.16*	−0.15*	−0.13*	−0.12*	0.10	−0.32
	(−0.26,−0.02)	(−3.61,−1.86)	(−0.28,−0.04)	(−0.26,−0.03)	(−0.25,−0.01)	(−0.24,−0.01)	(−0.02,0.22)	(−0.68,0.04)

^§^Eisenmann et al. [26] *p < 0.05, **p < 0.001. *HOMA-IR* Homeostasis Model Assessment for insulin resistance, *SBP* systolic blood pressure, *B* Beta, *CI* confidence interval

**Table 5:** Multivariate linear regression models with physical activity measures as independent variables and 13.5 year cardiometabolic risk factors as the dependent variables (*n* = 290)^†^

	13.5y Risk Factors							
	HOMA-IR	Fat %	Sum of Skinfolds (mm)	Waist Circumference (cm)	Total:HDL Cholesterol	Triglycerides	SBP (mmHg)	Metabolic Syndrome Score^§^
	B (95% CI)							
6–10 year Physical Activity								
Counts/day × 10^3^	−0.01	0.00	0.00	0.00	0.00	0.00	−0.01	−0.01
	(−0.02, 0.00)	(−0.01,0.01)	(− 0.01,0.01)	(− 0.01,0.01)	(− 0.01,0.01)	(− 0.01,0.01)	(− 0.02,0.00)	(− 0.01,0.02)
% time Moderate	− 0.05	− 0.08	0.02	0.01	0.01	0.01	− 0.02	0.05
	(− 0.12, 0.02)	(− 0.31,0.46)	(−0.04,0.08)	(− 0.05,0.06)	(− 0.06,0.06)	(− 0.06,0.07)	(− 0.08,0.04)	(− 0.23,0.13)
% time Vigorous	− 0.08	−0.11	− 0.05	− 0.05	−0.12	−0.10	− 0.08)	− 0.40*
	(− 0.22, 0.05)	(− 0.87,-0.66)	(− 0.17,0.06)	(− 0.16,0.06)	(− 0.24,0.02)	(− 0.23,0.03)	(− 0.20,0.04)	(− 0.75,0.05)
11–13 year Physical Activity								
Counts/day × 10^3^	−0.01*	−0.01*	−0.01	− 0.01	0.00	0.00	0.00	−0.02
	(−0.02,0.00)	(−0.01,−0.00)	(−0.02,0.00)	(− 0.02,0.00)	(− 0.01,0.01)	(− 0.01,0.01)	(−0.01,0.01)	(− 0.01,0.01)
% time Moderate	−0.04	−0.44*	−0.04	− 0.05*	−0.04	−0.02	0.01	−0.15*
	(−0.10,0.14)	(−0.77,−0.11)	(−0.09,0.01)	(− 0.10,0.00)	(− 0.09,0.02)	(− 0.08,0.03)	(−0.05,0.06)	(− 0.30,0.00)
% time Vigorous	−0.20*	−0.06*	−0.19*	− 0.17*	−0.15*	−0.13	0.20	−0.51*
	(−0.36,−0.03)	(−0.11,−0.01)	(−0.33,−0.05)	(− 0.30,−0.03)	(− 0.31,−0.00)	(− 0.29,−0.02)	(0.00,0.35)	(− 0.94,-0.08)

^†^Models adjusted for Age at activity assessment, sex, pubertal stage at 13.5 years, standard of living, maternal GDM status. ^§^Eisenmann et al. [26] *p < 0.05. *HOMA-IR* Homeostasis Model Assessment for insulin resistance *SBP* systolic blood pressure, *B* Beta, *CI* confidence interval

## Discussion

We aimed to study the effect of physical activity in childhood and early adolescence on risk factors for cardiometabolic disease at 13.5 years using longitudinal data from a South Indian cohort study [19]. We objectively measured the volume and intensity of physical activity at two time points and looked at tracking of physical activity from childhood to early adolescence. We were unable to make direct comparisons due to differences in ages, methodology and cut points used, but average accelerometer counts per minute were approximately 10–20% lower in our cohort than among children and adolescents of similar ages in Western countries [10, 32]. This supports findings from a global study of physical activity behavior which found Indian children to be among the least physically active in a comparison of 49 countries [18].

We found positive correlations between all of the physical activity variables measured at each of the two time points suggesting that physical activity tracks between childhood and early adolescence in this population. We also found that females were less physically active than males at both time points but particularly in early adolescence. Our data indicated that there may be an association between vigorous physical activity in childhood (6–10 years) and 13.5 year cardiometabolic disease risk based on a composite metabolic syndrome risk score. Vigorous physical activity at 11–13 years was associated with several risk factors for cardiometabolic disease as well as the composite risk score at 13.5 years. Total physical activity as measured by average total daily accelerometer counts may protect against insulin resistance and adiposity and moderate physical activity was associated with reduced fat percentage and waist circumference as well as the composite risk score. A study in the same cohort exploring associations of linear growth, fat gain and lean tissue gain during different age intervals in infancy and childhood with health-related outcomes at 13.5 years of age showed that faster fat gain in mid-late childhood predicts greater fat percentage and central adiposity, and higher systolic BP and insulin resistance (HOMA-IR) [33]. This fat gain may be a result of dietary exposures but may also be linked to less active lifestyles.

A recent meta-analysis of nine randomised controlled trials found that there was an overall decrease in insulin resistance among obese youth aged 6–18 years who were randomised to aerobic exercise interventions. A greater reduction in insulin resistance was observed among those aged 13–18 years than among those 6–12 years [34]. This finding supports our observation that physical activity in earlier childhood is less strongly associated with insulin resistance in adolescence whereas being physically active during early adolescence may be protective. Several cross-sectional studies have shown that greater physical activity is associated with reduced adiposity [35–37]. Evidence from a Mendelian Randomisation analysis indicated that increased adiposity causes reduced physical activity but this does not exclude reduced physical activity leading to increased adiposity [32].

Several studies have shown tracking of physical activity across the lifecourse [16, 38]. Although early life physical activity may not be necessary for prevention of later chronic disease, it may be habit-forming and thus children who are active are likely to go on to be active adolescents and adults. A recent study in India assessed the tracking of insulin resistance and measures of adiposity between the ages of 8 and 21 years in the Pune Children’s Study cohort [39]. It found that there was strong tracking of BMI, skinfolds and waist circumference and intermediate tracking of insulin resistance and so insulin resistance at 13.5 years is likely to be a risk factor for later diabetes.

The Identification and Prevention of Dietary and lifestyle-induced health Effects in Children and InfantS (IDEFICS) study evaluated cross-sectional associations between physical activity measured using actigraphs and clustering of cardiometabolic risk factors using a score comprised of systolic blood pressure, triglycerides, cholesterol, insulin resistance and cardiorespiratory fitness among 2104 children from 8 European countries (Italy, Estonia, Cyprus, Belgium, Sweden, Germany, Hungary, Spain) aged 6–9 years. Total physical activity and time spent moderately and vigorously active were all negatively correlated with the cardiometabolic risk score [10].

There are several potential mechanisms by which physical activity may reduce cardiometabolic risk. Absence of skeletal muscular contractions is associated with lower blood flow and therefore how efficient many metabolic processes are. For example, reduced transport of circulating glucose to muscle [40, 41]. There is evidence that moderate and vigorous physical activity are associated with increased uptake of glucose by skeletal muscle which leads to decreased blood glucose [42, 43]. It has also been proposed that lack of muscular activity turns off genes that maintain insulin sensitivity [44]. There is also evidence that lack of physical activity produces epigenetic changes including altered DNA methylation which are associated with increased cardiometabolic risk [45]. Furthermore physical activity reduces arterial stiffness and promotes vascular relaxation which may lead to a reduction in blood pressure [42]. In terms of lipid profile, there is evidence that physical activity upregulates enzymes that remove triglycerides and free cholesterol from the circulation and promotes production of HDL cholesterol [46].

It was beyond the scope of this work, but it is important to consider the effect of physical activity on fitness. In a prospective longitudinal study in Canada, participants were recruited at 9–15 years (*n* = 315) and physical activity was measured using accelerometers. Two years after recruitment, BMI, waist circumference, systolic blood pressure and cardiorespiratory fitness were measured. Time spent in vigorous physical activity was negatively associated with BMI and positively associated with fitness among all children [11]. Increased fitness may lead to increased tracking of vigorous physical activity so it may be that improving and maintaining fitness in early life is an important mechanism for reducing cardiometabolic risk.

### Strengths and limitations

The strengths of our study are that data were collected from a cohort which has been regularly followed up with low levels of participant attrition. We measured physical activity objectively in the majority of children within the cohort at two significant time points. This study was a secondary analysis of data collected as part of the birth cohort study and we therefore did not select the time points of measurement *a priori*. We plan to further assess cardiometabolic outcomes in late adolescence (20 years) in this cohort and look at the associations with physical activity.

A benefit of using accelerometers is that they are an objective method and not subject to recall bias and social desirability issues in the same way that questionnaires or diaries might be. When they are worn at the hip, accelerometers are designed to measure the volume of activities that involve body movements such as walking or running. They are less sensitive to activities that involve little vertical movement at the hip such as cycling, they are also not able to distinguish between movements on the flat versus those on a gradient and cannot be sensitive to activities that involve weights or resistance, for example carrying shopping [47]. It is also possible that there were activities that the children took part in that were not registered by the monitors because they were not worn at all times.

A limitation of our data is that we do not have energy intake at the time of the physical activity measurement so we were unable to adjust for this in our analysis. Energy intake may confound the association between physical activity and insulin resistance.

Furthermore, it is possible that the women and children are not representative of the population in this area. HMH is a private hospital and mainly caters to families from within the middle socioeconomic stratum of the Mysore population [19]. Therefore the majority of the women were literate and may have been better educated than the representative urban population of the region. However, the characteristics of the mothers during pregnancy and of the offspring at birth and during childhood are similar to those in other urban cohorts in South India [48–50].

### Public health implications

Our findings indicate that interventions are required to promote and increase physical activity among children and adolescents in India and this is supported by national level data [18, 51]. Implementing such interventions may help to prevent cardiometabolic disease and reduce the increasing health and human capital burden of these chronic conditions. Given our finding that females are less active, it is important that interventions are developed that take into account the challenges that are faced by females in India in this respect. A recent multi-centre study in India and sub-Saharan Africa led by the TALENT consortium (Transforming Adolescent Lives Through Nutrition) has been conducting qualitative research with adolescents and their caregivers to determine the barriers and facilitators to physical activity among this age group [52]. The ultimate aim of TALENT is to develop and implement interventions to increase activity in collaboration with adolescents and other stakeholders. It is likely that there will be some aspects of physical activity behaviour that can be addressed at the individual level. However, there are also likely to be cultural and structural issues such as pressure to excel academically and walkability difficulties in urban environments that will require a more systemic approach and political will to modify. At present the availability of data on such cultural and structural issues is limited in India [18, 51] and a combined approach of collecting such surveillance data and delivering interventions is required to address the rise in cardiometabolic disease.

## Conclusions

Our results indicate that total physical activity and particularly vigorous physical activity in childhood as well as early adolescence may be protective against the risk of cardiometabolic disease. The main finding of this study is that physical activity in early adolescence is associated with a more favorable body composition and cardiometabolic risk profile. To our knowledge this has not been demonstrated in a longitudinal study in India previously. We have shown that physical activity tracks from childhood to early adolescence and that females are less active than males. This finding should be taken into account when developing and implementing interventions. It is arguable that interventions are required at the individual, community and policy level to provide an enabling environment for physical activity and to instill physical activity as a routine behaviour throughout the lifecourse.

## Data Availability

The datasets generated and analysed during the current study are not publicly available due to Government of India regulations. However, the Parthenon Cohort team is open to data sharing with bona fide researchers on request subject to relevant Government of India permissions and ethics approval. For further information contact the corresponding author.

## Abbreviations

BF%: Body fat percentage
BMI: Body mass index
CPM: Counts per minute
CV: Coefficient of variation
CVD: Cardiovascular disease
ELISA: Enzyme-linked immunosorbent assay
HDL: High-density lipoprotein
HMH: Holdsworth Memorial Hospital
HOMA: Homeostatic model assessment
IQR: Inter-quartile range
LDL: Low-density lipoprotein
MUAC: Mid-upper arm circumference
NFHS: National Family Health Survey
PA: Physical activity
RT: Registered time

## Footnotes

1 Calculated using the formula (1/3 mean systolic bp) + (2/3 mean diastolic bp).
